# A mediation model of mindfulness and decentering: sequential psychological constructs or one and the same?

**DOI:** 10.1186/2050-7283-2-18

**Published:** 2014-07-07

**Authors:** Judith Gecht, Ramona Kessel, Thomas Forkmann, Siegfried Gauggel, Barbara Drueke, Anne Scherer, Verena Mainz

**Affiliations:** Department of Medical Psychology and Medical Sociology, RWTH Aachen University, Pauwelsstr. 19, 52074 Aachen, Germany; Institute of Psychology, RWTH Aachen University, Jägerstr. 17-19, 52066 Aachen, Germany

**Keywords:** Mindfulness, Decentering, Mechanism of change, Multiple mediation modeling, Structural equation modeling

## Abstract

**Background:**

Mindfulness and decentering are closely related processes both assumed to promote well-being. While some researchers claim that mindfulness and decentering can be clearly differentiated others suggest to use these concepts interchangeably. The precise relation between mindfulness and decentering remains unclear and therefore the present study aims to determine the relation between mindfulness and decentering.

**Methods:**

In a structural equation modeling framework, a mediation model was tested among a sample group of 495 university students (average age 20.8 years, 30.3% female).

**Results:**

The identified model shows an acceptable fit to the data and illustrates the role of decentering as a mediator of the relationship between mindfulness and depressive symptoms by complementary mediation and indirect-only mediation.

**Conclusion:**

The present results cannot sustain previous research, which converted mindfulness and decentering into one single variable. Rather the data suggests to treat mindfulness and decentering as two separable concepts and to regard decentering as an important working mechanism of mindfulness.

## Background

Mindfulness and its cultivation through the practice of meditation originated from ancient Eastern psychology and contemplative traditions, e.g., Buddhism, (Brown and Ryan 2003; Keng et al. 2011; Martin 1997). In these traditions, where conscious attention and awareness are actively cultivated, mindfulness meditation is described as a way of reducing mental suffering and encouraging the development of positive qualities, such as awareness, insight, and compassion (Kabat-Zinn 1990). Although mindfulness is an attribute of consciousness long believed to promote well-being, the incorporation of secular forms of mindfulness practice into contemporary Western medical and mental health care settings is quite recent (Baer 2010; Brown and Ryan 2003). In the past decades, traditional mindfulness meditation practices have been adapted and incorporated into several psychological interventions that are now widely available: e.g., Mindfulness-Based Stress-Reduction (MBSR; Kabat-Zinn 1982; Kabat-Zinn 1990), Dialectical Behavioral Therapy (DBT; Linehan 1993a; Linehan 1993b), Acceptance and Commitment Therapy (ACT; Hayes et al. 1999), Metacognitive Therapy (MCT; Wells 2000), or Mindfulness-Based Cognitive Therapy (MBCT; Segal et al. 2002).

In these contemporary Western interventions, the most commonly cited definition of mindfulness refers to mindfulness as the awareness that arises through “paying attention in a particular way: on purpose, in the present moment, and non-judgmentally” (Kabat-Zinn 1994, p. 4). Generally, based on this definition, mindfulness has been operationalized as a cognitive process of self-regulation of attention from a particular orientation towards one’s experience (Bishop et al. 2004). While “self-regulation of attention” refers to a non-elaborative observation and present-centered awareness of internal and external phenomena, “particular orientation” concerns an accepting attitude that people hold toward their thoughts and emotions, while experiencing these without maladaptive thought patterns like rumination (Bishop et al. 2004). These attributes of mindfulness are regarded as potentially effective antidotes against common forms of psychological distress because specific forms of self-focused attention can heighten or maintain psychopathology (Hayes and Feldman 2004), e.g., rumination (Nolen-Hoeksema 1991). Other modes of awareness lead to a more adaptive self-focused style, e.g., self-attentiveness motivated by curiosity (Trapnell and Campbell 1999).

However, even if the salutary effects of mindfulness-based interventions are widely accepted and their effectiveness has been demonstrated in numerous studies in clinical and non-clinical samples (Brown et al. 2007; Grossman et al. 2004; Keng et al. 2011), the underlying working mechanism by which the beneficial impact of mindfulness comes about seems less well understood. Shapiro and colleagues ( 2006) proposed a model of the mechanism of mindfulness and how mindfulness training may lead to positive outcomes, e.g., psychological symptom reduction. In their model they proposed that by cultivating mindfulness a shift in people’s perspective toward their inner experiences, i.e., their thoughts and emotions, is facilitated. They describe this shift as a change in relation to perceived mental and emotional experiences, which they term reperceiving, respectively, referring to it as decentering (Safran and Segal 1990; Shapiro et al. 2006). Decentering, then, is proposed to mediate the effect of mindfulness on subsequent mechanisms, e.g., values clarification or cognitive, emotional, and behavioral flexibility, which finally result in health benefits or may also be regarded as outcomes in themselves. In line with this model, several other authors suggested that mindfulness training increases metacognitive awareness, which has been defined as the ability to “reperceive” or to “decenter” from one’s thoughts and emotions (Bieling et al. 2012; Hargus et al. 2010; Orzech et al. 2009; Segal et al. 2002; Teasdale et al. 2002). The concept of decentering enables people to distance and disidentify themselves from the contents of their conscious thoughts and emotions (Safran and Segal 1990). By this, they gain a sense of mastery over their thoughts and emotions and feel able to perceive them as transient mental events, rather than to identify with them or to believe that thoughts and emotions are accurate reflections of the self or the reality (Safran and Segal 1990). It has been suggested, that a decentered perspective increases the range and adaptability of responses to both a stimulus cue and one’s impulse to react to that cue. Consequently, situational cues and responses can be addressed more consciously rather than to merely react to them in terms of habit or overlearned responses (Brown et al. 2007; Chambers et al. 2009; Shapiro et al. 2006; Teasdale et al. 1995). Accordingly, it is assumed that decentering enables people to alter the awareness of the relationship to as well as the frequency of their thoughts, which in turn improves people’s capacity to differentiate between an objective reality and a personally construed reality (Chambers et al. 2009; Safran and Segal 1990; Shapiro et al. 2006; Teasdale et al. 2000).

In the context of mood disorders, the decentered view of depression-related thoughts may enable individuals to prevent the escalation of or even reduce negative thinking patterns, e.g., rumination, and may offer some protection against relapse of major depression (Fresco et al. 2007b; Teasdale 1999). Consistent with the model of Shapiro and colleagues (Shapiro et al. 2006) it has been shown previously that training mindfulness enables individuals (1) to notice depressogenic thoughts and (2) to respond to them by redirecting attention to other aspects of the present moment, such as breathing, and in turn to disengage from depressive ruminative processes (Teasdale et al. 1995). The reduction in ruminative thinking that is predicted to occur with the adoption of a decentered perspective might explain why mindfulness training reduces the risk of relapse in recurrent major depression (Ramel et al. 2004; Teasdale et al. 2000; Teasdale et al. 2002). Moreover, recent studies also indicate that during MBCT a greater capacity to decenter may be fostered, which might protect against suicidal ideation and predict depressive symptoms at a 6-months follow-up (Bieling et al. 2012; Hargus et al. 2010).

The studies mentioned above regard mindfulness and decentering as two distinct concepts and report that decentering, or metacognitive awareness, can be increased by mindfulness training (e.g., Bieling et al. 2012; Hargus et al. 2010; Orzech et al. 2009; Teasdale et al. 2002). In addition to these studies, the model of mechanisms of mindfulness itself (Shapiro et al. 2006) has been empirically tested using mediation analysis in two studies, albeit, with conflicting findings (Carmody et al. 2009; Hayes-Skelton and Graham 2013). Hayes-Skelton and Graham ( 2013) have investigated the relationship between mindfulness, decentering, and social anxiety and found support for the model of Shapiro and colleagues ( 2006), indicating that decentering reflects a mechanism underlying the effect of mindfulness on social anxiety. Carmody and colleagues ( 2009) found disagreeing results compared to Shapiro and colleagues ( 2006) and Hayes-Skelton and Graham ( 2013) when assessing whether decentering acts like a key mechanism through which mindfulness relates to reductions in psychological symptoms. Thus, the mediating effect of decentering on the relationship of mindfulness and well-being was not supported by their results (Carmody et al. 2009). Instead, their results suggest to convert mindfulness and decentering into one single variable and to refer to them as one concept because the two variables represent two highly overlapping constructs (Carmody et al. 2009). In both studies, however, mindfulness and decentering were negatively correlated with good psychological well-being. Another approach to discuss the relationship of mindfulness and decentering was proposed by Wells and colleagues (Wells and Matthews 1994; Wells 2005) who introduced the concept of “detached mindfulness”. Detached mindfulness is referred to as a particular form of mindfulness that is made of different components. These components include, among others, characteristics of mindfulness, e.g., attentional detachment, as well as of decentering, e.g., comprehension of thoughts as events and not as facts (Wells 2005). Incorporating these features, detached mindfulness is antithetical to dysfunctional patterns of cognition like, e.g., perseverative thinking styles in the form of rumination.

From the above it may be apparent that mindfulness and decentering are closely related processes both believed to play a key role in accounting for the benefits of mindfulness-based interventions. However, the precise relation between mindfulness and decentering remains unclear because in the literature competing results have been reported. On the one hand, research has provided evidence that the two concepts can be clearly differentiated and arranged within a chain of sequential psychological processes (Hayes-Skelton and Graham 2013; Shapiro et al. 2006) wherein decentering represents a working mechanism, respectively, a mediator of mindfulness. On the other hand, some researchers claim that mindfulness and decentering refer to the same underlying concept and may be used interchangeably as it was shown in the study by Carmody and colleagues ( 2009). It is possible that the differences between these studies are due to distinct methodological procedures applied in the studies, e.g., different statistical approaches to mediation analysis or the operationalization of mindfulness and decentering.

The aim of the present research is to clarify the competing presumptions concerning the relationship between decentering and mindfulness. Expanding on former research in which mindfulness and decentering were treated as rather one-dimensional constructs (Carmody et al. 2009; Hayes-Skelton and Graham 2013), this study pays particular attention to the different facets of mindfulness and decentering and their underlying relationships. Because of this, we will try to elucidate specific aspects in the relationship between the two constructs in order to clarify, which subcomponents of mindfulness and decentering can be referred to as congruent and which of them can be clearly distinguished. In the context of a multiple mediation framework, combined with advanced strategies to estimate the magnitude of the mediated effect for each path in the mediation model (Fairchild et al. 2009), we aim to identify whether aspects of decentering influence the effect of mindfulness on symptoms of depression. More specifically, in the present study we will investigate whether (a) decentering mediates the salutary effect of mindfulness on symptoms of depression or whether (b) the effect of decentering and mindfulness is congruent, because the concepts share enough variance with each other as to conclude that they are one and the same. We will relate the effects of both variables to symptoms of depression because the effects of decentering were most often investigated in relation to this syndrome.

## Methods

### Study design and sample

To investigate the hypothesized relationships between mindfulness, decentering, and depressive symptoms, a cross-sectional questionnaire-based study among a mixed sample of undergraduate university students was conducted. The questionnaires were filled out anonymously in the context of university lectures. Before the start of the lecture a questionnaire was handed out to every student entering the lecture hall. The students returned the completed questionnaires directly after the lecture when leaving the lecture hall. By this procedure we were able to ensure that all eligible students had the possibility to participate in the study as well as to check the response rate. Students were informed about the content of the research project and participated voluntarily. For their participation the students received a small token. The study procedures were approved by the local ethics committee of the Medical faculty of RWTH Aachen University (EK148/11). The present study is a secondary analysis of data published elsewhere (Gecht et al. 2014).

### Self-report measures

#### Mindfulness

The Kentucky Inventory of Mindfulness Skills (KIMS; Baer et al. 2004) measures the presence of mindful skills in daily life. In the present study, the German form of the short version of the KIMS was used (KIMS-Short; Hoefling et al. 2011), which is a 20-item instrument designed to measure five skills of mindfulness: Describing (DES, 5 items, e.g., “I’m good at finding words to describe my feelings”), Accepting without Judgment (AWJ, 5 items, e.g., “I make judgments about whether my thoughts are good or bad”), Acting with Awareness (AWA, 4 items, e.g., “When I do things, I get totally wrapped up in them and don’t think about anything else”), Observing of internal phenomena (OBS-IN, 3 items, e.g., “When I’m walking, I deliberately notice the sensations of my body moving”), and Observing of external phenomena (OBS-OUT, 3 items, e.g., “I pay attention to sounds, such as clocks ticking, birds chirping, or cars passing”). Items are rated on a 5-point Likert-scale ranging from “never or very rarely true” (1) to “always or almost always true” (5). A mean score per scale is calculated ranging from 1 to 5, with higher scores indicating the presence of more mindful skills. Internal consistencies, indicated by Cronbach’s alpha, for the different subscales range from .70 for AWA to .82 for DES in the present study.

#### Decentering

The German version of the Experiences Questionnaire (EQ-D; Gecht et al. 2014), originally developed by Fresco and colleagues ( 2007a), was used to measure the participants’ capacity to decenter. The EQ-D encompasses two subdimensions of decentering: Accepting Self-Perception (ASP; 4 items, e.g., “I am able to accept myself as I am”) and Distanced Perspective (DP; 4 items, e.g., “I can separate myself from my thoughts and feelings”). Responses are indicated on a 5-point Likert-scale ranging from “never” (0) to “all the time” (4). Per subscale, total scores are calculated that can range from 0 to 16, with higher scores indicating a greater capacity to decenter. The EQ-D shows generalizability across both genders and different age groups, and preliminary results support its construct validity. In a previous report (Gecht et al. 2014), the construct reliabilities for both decentering factors exceeded the threshold for good fit of ≥ .70 (Hair et al. 2010, p. 709).

#### Depression

The Rasch-based Depression Screening (DESC; Forkmann et al. 2009; Forkmann et al. 2010) was developed as an one-dimensional measure to screen for depression in patients suffering from mental and somatic disorders. The 10 items of the DESC refer to the last two weeks. Participants are asked to indicate how often they experienced each symptom on a 5-point Likert-scale ranging from “never” (0) to “always” (4) with higher scores indicating more symptoms of depression and total scores of ≥ 12 suggesting the presence of a depressive episode (Forkmann et al. 2009; Vehren et al. 2013).

### Procedure

#### Data analysis

Descriptive statistics for the study sample and the study variables were calculated with SPSS 20 (IBM 2011). A *t*-test in combination with the Bonferroni correction was run in order to examine the mean values of the study variables for statistically significant differences. By inspecting the Pearson’s correlation coefficients, *r,* the relationships between mindfulness and decentering factors were examined. Correlations between mindfulness and decentering variables exceeding the value of .80 indicate that one should refer to them as a single factor (Bühner 2011).

In order to estimate the paths in the mediation model, a multiple mediation analysis (MacKinnon et al. 2007) was performed in a structural equation modeling (SEM) context using M*plus*® Version 6 (Muthén and Muthén 2010). SEM is a multivariate technique that combines the properties of factor analysis, regression analysis, and path analysis. SEM can estimate complex model structures while accounting for multiple influences, which may simultaneously affect the outcome variable (Hair et al. 2010). In mediation analysis, different hypothesized associations between variables are dissected into components in order to reveal a possible causality. Although causal inferences cannot be established definitely in correlation analysis, mediation analysis can provide evidence that one path is more probable than another (Shrout and Bolger 2002).

The different effect-size measures are calculated using the SPSS macro provided by Fairchild and colleagues (2009).

#### Model fit

In the multiple-mediator model, the mediators Accepting Self-Perception and Distanced Perspective were allowed to covary, as were the residual variances of the independent mindfulness factors. Preacher and Hayes ( 2008) have recommended that residuals associated with the mediators be permitted to covary, because fixing these parameters to zero would imply that any covariance among the decentering factors is completely due to the effects of the mindfulness factors. The appropriateness of the mediation model was assessed with global goodness-of-fit indices. These fit indices portray the degree to which the proposed model adequately represents the empirical associations. Three indices were employed: the Root-Mean Square Error of Approximation (RMSEA), the Bentler Comparative Fit Index (CFI), and the Tucker-Lewis Index (TLI). RMSEA-values ≤ .07 in combination with a value for CFI or TLI ≥ .90 suggest an acceptable model fit (Hair et al., 2010). Additional support for the identified RMSEA-value would be evidenced by a 90% confidence interval (CI) of the RMSEA including the .05-value and not exceeding an upper limit of .10 (Brown 2006).

#### Quantification of the effects in the mediation model

Different effects are tested in the present analysis to identify mediation respectively non-mediation (Preacher and Hayes 2008). Firstly, the *direct effects* of the mindfulness factors on the decentering factors were estimated as were the direct effects of the mindfulness factors and decentering factors on symptoms of depression. Secondly, *specific indirect effects* are calculated, defined as the indirect effect of the independent variable *X* (i.e., a mindfulness subscale) via a mediator *M* (i.e., a decentering factor) on the dependent variable *Y* (i.e., symptoms of depression). Thirdly, the *total indirect effects*, defined as the sum of the specific indirect effects, and finally, the *total effects* are estimated, which represent the sum of the direct effect of one mindfulness subscale on depressive symptoms and its corresponding specific indirect effects.

The different effects and their corresponding 95% CIs were calculated with M*plus* because of its capability to estimate both total and specific indirect effects for multiple mediator models, using bootstrapping and providing bias-corrected (BC) 95% CIs (Preacher and Hayes 2008). Simulation research (Fritz and MacKinnon 2007; MacKinnon et al. 2002; MacKinnon et al. 2004; Williams and MacKinnon 2008) has shown that BC bootstrapping is a more valid and powerful method for testing intervening variable effects than the “causal steps approach” (Baron and Kenny 1986) and the “Sobel test” (Sobel 1982). Bootstrapping is a resampling method, which is conducted by randomly sampling, with replacement, cases from the original sample of *N* observations so that a new sample of *N* observations is build. With every bootstrap draw, sample statistics, such as direct and indirect effects, are calculated. Upon completion of the bootstrapping process, the distribution of these estimates function as an empirical approximation of the sampling distribution of the indirect effect. In the present study, the number of bootstrap draws specified was 5,000 as recommended by Hayes ( 2009). Based on the size of the estimates of the different effects, their corresponding BC 95% CIs can be generated: if zero is not between the lower and upper bound, the effect is not zero with 95% confidence.

#### Classifying the type of mediation and R^2^ effect sizes for the mediated effects

Based on the identified effects, obtained by the method recommended by Preacher and Hayes ( 2008) as described above, Zhao and colleagues ( 2010) provide a step-by-step procedure for classifying the type of mediation and interpreting the implications of the findings. In the first step, the indirect effect (*Mi*) is inspected for significance to determine whether mediation (in case of significance) or non-mediation (in case of non-significance) is present. In the second step, in order to classify the type of mediation or non-mediation, it is determined whether the direct effect of the respective mindfulness subscale (*Xi*) on depression symptoms (*Y*) is significant. By this approach three patterns consistent with mediation, i.e., 1 to 3, and two patterns consistent with non-mediation, i.e., 4 and 5, can be identified (Zhao et al. 2010):

1. Complementary mediation: the indirect effect and the direct effect are both significant and the multiplication of their coefficients is positive.
2. Competitive mediation: the indirect effect and the direct effect are both significant and the multiplication of their coefficients is negative.
3. Indirect-only mediation: the indirect effect is significant, but the direct effect is not.
4. Direct-only non-mediation: the indirect effect is not significant, but the direct effect is.
5. No-effect non-mediation: neither the direct effect nor the indirect effect is significant.

The practical utility of the hypothesized mediators in a model is identified by estimating effect-size measures that compare the magnitude of different effects in the model. Recently, Fairchild and colleagues ( 2009) have introduced a measure of the effect-size that reflects the proportion of the variance in *Y* explained by the indirect effect. They propose different *R*^2^ effect-size measures for the inspection of the relative contribution of individual paths in the mediation model, as well as the unique variance in an outcome variable *Y* that is explained by a mediated effect, *R*^2^_med_. The *R*^2^_med_ provides information about the extent to which *X* predicts a variance in *M*, which subsequently predicts a variance in *Y*, thus the variance in the dependent variable *Y* explained by the independent variable *X*, and the mediator *M* variable together. Hence, the mediated effect, *R*^2^_med_, contains information about the practical significance of the overall mediation relation. Because the computation of *R*^2^_med_ is based on estimating differences between the individual components of the mediation model it is possible to obtain negative values for *R*^2^_med_. Such a negative value of the *R*^2^_med_ estimate would point to the presence of a suppression effect: the variance in the outcome variable predicted by a pair or a group of variables may be reduced as compared to a prediction from either variable alone (Preacher and Kelley 2011).

Dividing *R*^2^_med_ by the overall *R*^2^, i.e., *R*^2^_*Y*, *MX*_, estimates the effect-ratio, which represents the proportion of the total effect of *X* on *Y* that is mediated by *M*. Furthermore, component *r*^2^ measures for mediation complement the results from the *R*^2^_med_. These are referred to as partial correlations, namely, *r*^2^_*XY .M*_, as the squared partial correlation of *Y* and *X,* partialed for the influence of the mediator *M*, and *r*^2^_*MY .X*_ representing the squared partialed correlation of *Y* and *M,* partialed for *X*.

## Results

### Sample descriptive data

From the total sample (N = 565, one student refused to participate) 70 cases were excluded due to missing values on items of the EQ-D, the KIMS- Short, or the DESC. The present analysis is based on 495 undergraduate students (30.3% female) with an average age of 20.8 years (standard deviation = 1.9; range = 18 – 33 years). Forty-eight participants (9.7%) scored above the cut-off level ≥ 12 on the depression measure, pointing to the possible presence of a depressive episode (Forkmann et al. 2009). Because of the anonymity of the data collection these students could not be referred to a therapist. However, the results of our study, regarding sample descriptive data, have been communicated to the professor, who gave us the opportunity to collect the data in his lectures and who is responsible for the students in question. Furthermore, all students were informed that within the RWTH Aachen University Hospital the “Center for Mental Health for Undergraduate and Postgraduate Students (ZPG)” gives students the possibility to ask for support if they suffer from emotional crisis or psychological problems. Regarding the ethnical background, most participants were Caucasian. Only eight students (1.6%) indicated that they do not to speak German as their first language and that they have lived in Germany for less than 5 years. Based on the Mahalanobis distance statistic (Kline 2011) none of these participants was identified as an outlier on any of the variables in the mediation model. Table 1 displays the descriptive data for the study variables and the correlation matrix between the examined variables.

**Table 1:** **Descriptive statistics for the study variables**

Factor	Mean (SD ^a^)	Cronbach’s alpha ^b^	1	2	3	4	5	6	7
DESC^c^	4.9 (4.9)	.88							
Decentering^d^									
ASP	11.9 (2.5)	.70	-.42**						
DP	8.7 (2.7)	.70	-.41**	.39**					
Mindfulness^e^									
AWJ	3.7 (0.8)	.79	-.48**	.33**	.35**				
DES	3.5 (0.8)	.82	-.29**	.32**	.21**	.17**			
AWA	2.9 (0.7)	.70	-.09	.20**	.18**	.01	.08		
OBS-IN	3.0 (0.9)	.69	-.02	.17**	.04	.11*	-.06	.12**	
OBS-OUT	3.2 (0.8)	.62	-.03	.12**	.03	.13**	-.09*	.03	.56**

Note. ^a^SD: standard deviation; ^b^reliability estimation; ^c^DESC: Rasch-based Depression Screening (Forkmann et al. 2009); ^d^EQ-D (Gecht et al. 2014): ASP = Accepting Self-Perception; DP = Distanced Perspective; ^e^KIMS-Short (Hoefling et al. 2011): AWJ = Accepting without Judgment; DES = Describing; AWA = Acting with Awareness; OBS-IN = Observing of internal phenomena; OBS-OUT = Observing of external phenomena. *The correlation is significantly different from zero at the .05 level (two-tailed); **The correlation is significantly different from zero at the .01 level (two-tailed).

Regarding decentering, participants reported to engage more in Accepting Self-Perception (mean = 11.9, standard deviation (*SD)* = 2.5) than in Distanced Perspective (mean = 8.7, *SD* = 2.7). Related to mindfulness, participants reported mainly to engage in Accepting without Judgment (mean = 3.7, *SD* = 0.8) and less in Accepting with Awareness (mean = 2.9, *SD* = 0.7) or Observing of internal phenomena (mean = 3.0, *SD* = 0.9). Results of the Bonferroni adjusted *t*-test revealed significant differences (*p* < .001) for all studied variables besides for the comparison between Accepting without Judgment and Observing of internal phenomena (*p* = .144). The strongest correlations between the decentering and the mindfulness factors were found for Distanced Perspective and Accepting without Judgment (*r* = .35), followed by Accepting Self-Perception and Accepting without Judgment (*r* = .33). The two decentering factors were correlated with *r* = .39 while the strongest correlation between mindfulness factors was found for the two Observing factors (*r* = .56). All these correlations were significant at α ≤ .01.

### Procedure

#### Model fit

**Figure 1:**
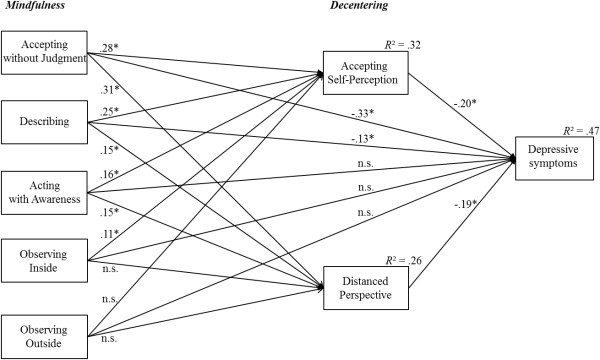
The multiple mediation model of mindfulness, decentering and depressive symptoms with standardized direct effects; * ***p*** **< .05; n.s. = not significant.**

The mediation model displayed in Figure 1 showed an acceptable fit to the empirical data according to measures of the global fit. The RMSEA equaled to .042 with a corresponding 90% CI, ranging from .038 to .046. The CFI was .914 and the TLI was .904. The total explained variance in depression scores by accounting for all used variables in the model equaled to .47. The variance explained in Accepting Self-Perception and Distanced Perspective by the mindfulness factors was .32 and .26, respectively.

#### Quantification of the effects in the mediation model

The unstandardized direct, specific indirect, total indirect, and total effects along with their corresponding standard error and *z*-scores are shown in Table 2. For reasons of comparability, the standardized effects are also included in the last column of Table 2. Figure 1 visualizes the standardized direct effects.

**Table 2:** **Unstandardized estimates b and standardized effects β in the mediation model**

Type of effect	***b*** ^a^	***SE*** ^b^	***z*** ^c^	BC 95% CI [LO – HO] ^d^	β ^e^
*Direct effects*					
AWJ→ASP	.922*	.153	6.034	[.619; 1.216]	.281*
AWJ→DP	1.110*	.167	6.657	[.780; 1.429]	.311*
AWJ→DEP	-2.101*	.259	-8.108	[-2.615; -1.595]	-.329*
DES→ASP	.835*	.151	5.532	[.545; 1.129]	.250*
DES→DP	.552*	.162	3.419	[.235; . 862]	.152*
DES→DEP	-.813*	.318	-2.559	[-1.486; -.246]	-.125*
AWA→ASP	.600*	.162	3.714	[.274; .915]	.159*
AWA→DP	.605*	.192	3.157	[.215; .968]	.148*
AWA→DEP	.082	.285	.288	[-.470; .650]	.011
OBS-IN→ASP	.300*	.131	2.291	[.042; .559]	.111*
OBS-IN→DP	.034	.155	.216	[-.277; .336]	.011
OBS-IN→DEP	.110	.233	.470	[-.332; .582]	.021
OBS-OUT→ASP	.143	.154	.927	[-.160; .445]	.045
OBS-OUT→DP	.093	.194	.479	[-.272; .480]	.027
OBS-OUT→DEP	-.135	.269	-.499	[-.654; .387]	-.022
ASP→DEP	-.386*	.107	-3.593	[-.594; -.179]	-.198*
DP→DEP	-.346*	.078	-4.451	[-.508; -.204]	-.194*
*Specific indirect effects*					
AWJ→ASP→DEP	-.356*	.122	-2.908	[-.649; -.160]	-.056*
AWJ→DP→DEP	-.384*	.104	-3.708	[-.631; -.217]	-.060*
DES→ASP→DEP	-.322*	0.116	-2.782	[-.603; -.134]	-.050*
DES→DP→DEP	-.191*	.074	-2.580	[-.373; -.076]	-.029*
AWA→ASP→DEP	-.232*	.092	-2.526	[-.458; -.088]	-.032*
AWA→DP→DEP	-.210*	.082	-2.564	[-.402; -.079]	-.029*
OBS-IN→ ASP→DEP	-.116*	.061	-1.898	[-.271; -.022]	-.022*
OBS-IN→ DP→DEP	-.012	.055	-.211	[-.129; .095]	-.002
OBS-OUT→ ASP→DEP	-.055	.063	-.877	[-.201; .052]	-.009
OBS-OUT→ DP→DEP	-.032	.069	-.469	[-.180; .093]	-.005
*Total indirect effects*					
AWJ→ASP, DP→DEP	-.740*	.178	-4.166	[-1.146; -.443]	-.116*
DES→ASP, DP→DEP	-.513*	.179	-3.457	[-.848; -.263]	-.079*
AWA→ASP, DP→DEP	-.441*	.130	-3.392	[-.756; -.232]	-.060*
OBS-IN→ASP, DP→DEP	-.127	.091	-1.398	[-.341; .027]	-.024
OBS-OUT→ASP, DP→DEP	-.087	.108	-.811	[-.318; .104]	-.014
*Total effects*					
AWJ→ASP, DP→DEP; AWJ→DEP	-2.841*	.312	-9.112	[-3.447; -2.227]	-.445*
DES→ASP, DP→DEP; DES→DEP	-1.326*	.316	-4.202	[-1.958; -.729]	-.204*
AWA→ASP, DP→DEP; AWA→DEP	-.359	.308	-1.164	[-.970; .252]	-.060
OBS-IN→ASP, DP→DEP; OBS-IN→DEP	-.018	.244	-.073	[-.479; .483]	-.003
OBS-OUT→ ASP, DP→DEP; OBS-OUT→DEP	-.222	.283	-.785	[-.783; .322]	-.036

*Note.* ^a^Unstandardized coefficients; ^b^Standard error; ^c^ *z*-value; ^d^Lower and upper bound of bias-corrected 95% confidence interval with 5,000 bootstrap samples; ^e^Standardized coefficients; AWJ = Accepting without Judgment; ASP = Accepting Self-Perception; DP = Distanced Perspective; DEP: depressive symptoms; DES = Describing; AWA = Acting with Awareness; OBS-IN = Observing of internal phenomena; OBS-OUT = Observing of external phenomena. *p < .05.

Accepting without Judgment and Describing showed significant direct, specific indirect, total indirect, and total effects (all *p* < .05). In contrast, related to Acting with Awareness and Observing of internal phenomena several differences among the various effects were found. Acting with Awareness displayed significant specific indirect effects, total indirect effects and direct effects on Accepting Self-Perception and Distanced Perspective (all *p* < .05), whereas non-significant scores were found for the direct effect on symptoms of depression and the total effect. Regarding Observing of internal phenomena, significant effects were only found for the direct effect on Accepting Self-Perception and the specific indirect effect on depressive symptoms via Accepting Self-Perception (all *p* < .05). No significant effects were found for Observing of external phenomena. Both direct effects of the mediators Accepting Self-Perception and Distanced Perspective on depressive symptoms were significant (*p* < .05).

Accepting without Judgment showed the largest total effect (β = -.445). Among the direct effects, the largest effects were found for Accepting without Judgment on depression scores (β = -.329), for Accepting Self-Perception (β = .281), and for Distanced Perspective (β = .311). The specific indirect effects were strongest for Accepting without Judgment via Distanced Perspective on depressive symptoms (β = -.060) and the total indirect effect was most strongly represented by Accepting without Judgment (β = -.116).

#### Classifying the type of mediation and R^2^ effect sizes for the mediated effects

Applying the step-by-step procedure recommended by Zhao and colleagues ( 2010) complementary mediation, indirect-only mediation, and no-effect non-mediation were identified. Complementary mediation was revealed for Describing and Accepting without Judgment via both decentering factors and for the specific indirect as well as for the total indirect effects. Indirect-only mediation was established for Acting with Awareness as specific indirect effect through both decentering factors as well as for the total indirect effect. Observing of internal phenomena showed indirect-only mediation for the specific indirect effect through Accepting Self-Perception. No-effect non-mediation was identified for the specific indirect effect of Observing of internal phenomena via Distanced Perspective, for Observing of external phenomena via both decentering variables, and for the total indirect effect of both observation variables. Table 3 displays complementary and indirect-only mediation for the different specific indirect and total indirect effects along with the corresponding *R*^2^ effect-sizes.

**Table 3:** **Classification of the mediation and R** ^**2**^ **effect-size measures**

Type of Mediation	Overall ***R*** ^2^	***R*** ^2^_med_^a^	***r*** ^2^_***XY .M***_^b^	***r*** ^2^_***MY .X***_^c^	Effect-ratio
*Complementary mediation*					
Specific indirect					
AWJ→ASP→DEP	.307	.098	.161	.099	.319
AWJ→DP→DEP	.298	.100	.158	.087	.336
DES→ASP→DEP	.201	.056	.032	.129	.279
DES→DP→DEP	.209	.040	.050	.138	.191
Total indirect					
AWJ→ASP, DP→DEP	.339	.137	.125	.140	.403
DES→ASP, DP→DEP	.264	.063	.026	.198	.238
*Indirect-only mediation*					
Specific indirect					
AWA→ASP→DEP	.174	.007	.000	.168	.040
AWA→DP→DEP	.167	.007	.000	.161	.042
OBS-IN→ASP→DEP	.176	-.001	.002	.176	-.006
Total indirect					
AWA→ASP, DP→DEP	.245	.007	.001	.239	.028

Note. ^a^ Variance explained by the mediated effect; ^b^ squared partial correlation of Y and X partialed for mediator M; ^c^ squared partialed correlation of Y and M partialed for X; symptoms of depression (DEP); Accepting Self-Perception (ASP); Distanced Perspective (DP); Describing (DES); Accepting without Judgment (AWJ); Acting with Awareness (AWA); Observing of internal phenomena (OBS-IN); Observing of external phenomena (OBS-OUT).

##### Complementary mediation

The *specific indirect effects* of Accepting without Judgment and Accepting Self-Perception as well as Distanced Perspective accounted each for approximately 30% of the variance in depressive symptoms. The proportion of variance in depression scores that was explained by the mediated effects equaled nearly 10% via Accepting Self-Perception as well as Distanced Perspective. This indicated that 10% of the variance of Accepting without Judgment in explaining depressive symptoms was attributable to the indirect effects via Accepting Self-Perception, respectively Distanced Perspective. The proportion of variance in depressive symptoms explained by the partialed effect of Accepting without Judgment, and thus the sole contribution of Accepting without Judgment without the influence of decentering, equaled nearly 16%. In turn, partialed effects of Accepting Self-Perception and Distanced Perspective, besides the effects of Accepting without Judgment, accounted for 10% in depression scores. Overall, the estimation of the effect-ratio revealed that the mediated effect accounted for 32% (.098/.307 for Accepting Self-Perception) to 34% (.100/.298 for Distanced Perspective) of the explained variance.

Regarding the indirect specific effect of Describing via Accepting Self-Perception, the *R*^2^_med_ value of .056 indicated that slightly less than 6% of the variance in the depression score was attributable to this indirect effect. Considering that approximately 20% (overall *R*^2^ = .201) of the participants’ depression scores was explained, it followed that 27.9% (.056/.201) of the explained variance in the model was due to the mediated effect, respectively due to the introduction of Accepting Self-Perception into the relation of Describing and depression scores. For the mediation by Distanced Perspective, it was revealed that from the variance in depression 4% was attributable to the common variance of Describing and Distanced Perspective. Overall, 20.9% was explained, pointing out that 19.1% (.040/.209) of the explained variance in depression scores was attributable to the common variance of Describing and Distanced Perspective.

Overall, the *total indirect effect* of Accepting without Judgment and both decentering variables together explained approximately 34% of the variance in depressive symptoms. The *R*^2^_med_ value of .137 indicated that nearly 14% of the variance in the depression score was attributable to the indirect effect. It followed that 40.3% (.137/.339) of the explained variance in the model was due to the mediated effect, respectively due to the introduction of the decentering factors into the relation of Accepting without Judgment and depression scores. Describing and the total indirect effect from both decentering variables together explained 26.4% of the variance in depression scores, while the proportion of variance that was attributable to the mediated effect amounted to 6.3%, indicating that 23.8% (.063/.264) of the explained variance was accounted for by the mediated effect.

Inspecting the contribution of the individual effects, among the complementary mediation the weakest paths were identified for Describing on depressive symptoms, partialed for the total effect of the mediating variables (*r*^2^_*XY .M*_ = .026). The strongest effect was obtained for the total effect of the mediating variables, partialed for the influence of Describing (*r*^2^_*MY .X*_ = .198). Regarding the specific indirect effects, the weakest effect was found for Describing on depressive symptoms (*r*^2^_*XY .M*_ = .032) and the strongest path was identified for Accepting without Judgment on depressive symptoms (*r*^2^_*XY .M*_ = .161), both partialed for the influence of Accepting Self-Perception.

##### Indirect-only mediation

The total indirect effect of Acting with Awareness explained 24.5% of the overall variance in depressive symptoms, from which less than 1% was due to the mediated effect, leading to an effect-ratio of .028 (.007/.245). The correlation of Acting with Awareness with depressive symptoms partialed for the effect of the mediators was .001 while the correlations of the mediators with depressive symptoms partialed for the effect of Acting with Awareness equaled .239.

An examination of the individual contributions of the component paths indicated that regarding the specific effects the weakest path in the model was found for the relation between Acting with Awareness and depressive symptoms partialed for Accepting Self-Perception as well as for Distanced Perspective (*r*^2^_*XY .M*_ = .000, respectively). The strongest path in the model was represented by the relation between Accepting Self-Perception on depressive symptoms partialed for the influence of Observing of internal phenomena (*r*^2^_*MY ..X*_ = .176). Among the total indirect effects, the weakest paths were found for Acting with Awareness partialed for the mediating variables (*r*^2^_*XY .M*_ = .001, respectively), while the effects of the mediating variables together were strongest when partialed for Acting with Awareness (*r*^2^_*MY ..X*_ = .239).

## Discussion

The main motivation of the present study was to investigate whether decentering and mindfulness are two concepts referring to the same underlying construct as proposed by Carmody and colleagues ( 2009) or whether they reflect two distinct concepts as emphasized by other authors (Hayes-Skelton and Graham 2013; Shapiro et al. 2006). The present analysis did not reveal any correlation exceeding the critical value of .80 between a mindfulness skill and the capacity to decenter, which would be necessary to conclude that mindfulness and decentering share enough variance as to refer to them as indicators of the same underlying construct and to aggregate them into a single variable (Bühner 2011). Furthermore, all mindfulness skills together explain 32% of the variance in Accepting Self-Perception and 26% of the variance in Distanced Perspective. The common variance of decentering and depressive symptoms without the influence of mindfulness ranges from 9% up to 24%. These findings imply that the present data cannot support previous research referring to decentering and mindfulness as one single construct (Carmody et al. 2009). Instead, the present findings yield evidence for supporting the hypothesis that decentering and mindfulness represent two separate concepts and furthermore that decentering mediates the relationship between mindfulness skills and the severity of depressive symptoms (Shapiro et al., 2006). Additionally, the fact that decentering and mindfulness were identified to be two different constructs is underpinning the notion that detached mindfulness (Wells 2005) is a multifaceted construct consisting of distinguishable and interrelated components. In the present model, 47% of the variance in depression scores is explained by mindfulness and decentering variables together. Taking into account Shapiro and colleagues’ (2006) suggestion that decentering is only one of the working mechanisms through which mindfulness excerpts its effect on depressive symptoms, decentering is shown in the present study to be an important factor in transmitting the beneficial effects of mindfulness.

The present study also yields the important result that none of the mindfulness skills has an influence on depressive symptoms without the influence of decentering, exemplified by the absence of direct-only non-mediation. In contrast, complementary and indirect-only mediation were identified, indicating that the effect of mindfulness on depressive symptoms is mediated by the influence of decentering. This finding suggests that we have to regard decentering as a working mechanism of mindfulness.

By identifying complementary mediation in the present study, it was shown that even if no exclusive effect of mindfulness on depressive symptoms can be found, the combined effect of decentering and mindfulness on depressive symptoms has an important contribution. Accepting without Judgment and Describing exert their influence on depressive symptoms through two distinct pathways: directly from the respective mindfulness skill itself and indirectly through decentering. This result indicates that decentering seems to be an important mediator for the relationship between depressive symptoms and the ability to be non-judgmental about one’s present-moment experiences (AWJ) as well as to describe and label observed thoughts and feelings (DES). The influence of Accepting without Judgment on depressive symptoms seems to be stronger than the influence of Describing as indicated by stronger indirect and direct effects of Accepting without Judgment. While the effect of Accepting without Judgment is stronger related to depressive symptoms than to the decentering variables, it is the other way around for Describing, which is stronger related to decentering than to symptoms of depression. The effect–ratios that are higher for Accepting without Judgment than for Describing also underline this result. These results point toward regarding decentering as a stronger mediator for the effects of people’s ability to accept present-moment experiences non-judgmentally (AWJ) than for the effects of the ability to label thoughts and emotions (DES).

However, the mediating effect of decentering is not only exemplified in the present study in combination with direct effects of mindfulness. Indirect-only mediation was identified for two mindfulness skills to act with awareness by fully engaging in one’s current activity (AWA) and to observe bodily sensations, thoughts and emotions (OBS-IN). The identification of indirect-only mediation for these skills indicates that their influence on depressive symptoms is only effective through their effects on decentering. This means, significant direct effects from Acting with Awareness and Observing of internal phenomena on depressive symptoms could not be demonstrated, but their influence on depressive symptoms was evidenced by the indirect paths through the mediating decentering variables Accepting Self-Perception and Distanced Perspective. Thus, the extent to which a person engages in the respective mindful skill does not directly minimize depressive symptoms but exerts an influence on the amount to which the person engages in decentering, which, in turn, influences the severity of depressive symptoms. When inspecting Table 3 it becomes apparent that the proportion of variance explained by the mediated effect was below 1% for Acting with Awareness and Observing of internal phenomena and that the corresponding effect-ratios were also very low. However, effect-ratios are less informative for indirect-only mediation because the direct effect of the independent variable is per definition non-significant and therefore small (Zhao et al. 2010), following that most of the variance in the dependent variable is explained by the mediating variable alone. Therefore, it is advised to refer to the overall *R*^2^ for drawing conclusions about the magnitude of the mediation effect. In the present study, the associations underlying this measure are all of comparable magnitude. This indicates that both decentering variables mediate the effects of Acting with Awareness and the Observation of inner experiences on depressive symptoms equally strong, i.e., around 17% of the variance in depressive symptoms is explained by the mediation.

Inspecting the component *r*^2^ measures as well as the direct effects, specific relationships between the different variables in the mediation model can be interpreted uniquely. This information helps to improve the understanding of the relationship between mindfulness, decentering, and depressive symptoms. In general, mindfulness and decentering were positively correlated with each other. Regarding the relations between mindfulness skills and decentering with symptoms of depression negative associations were identified. Interestingly, positive, but however not significant, direct effects were found for focusing with awareness on one’s current activity (AWA) and paying careful attention to inner phenomena (OBS-IN) on depression. Further inspection of the component paths reveals that the strongest links in the model are those paths, which include the skill to accept present-moment experiences without judgment (AWJ), both directly and indirectly via decentering. This means, participants, who were more able to refrain from the evaluation of present-moment experience (AWJ), show lower scores on the depression measure. At the same time, they have a more accepting attitude toward themselves (ASP) and refer to their thoughts and feelings from an objective perspective (DP). The weakest links in the model are those relations, which include the observation of external phenomena (OBS-OUT) followed by the observation of internal phenomena (OBS-IN). Examination of the *R*^2^_med_ value for the specific indirect path of Observing of internal phenomena via Accepting Self-Perception on depression reveals a negative value, which suggests that a suppression effect may be present. However, the direct effect of the observation of internal phenomena (OBS-IN) on depression was not significant and, therefore, competitive mediation was not established, which could have supported the presence of suppression. Instead, no-effect non-mediation was identified for the specific indirect effect of Observing of internal phenomena via Distanced Perspective, for Observing of external phenomena via both decentering variables, and for the total indirect effect of both observation variables. The non-significance among the observation factors could arise due to a power problem, which hinders the detection of significant effects on the side of the observing subscales. These subscales are only composed of three indicators each and show low reliability coefficients accordingly. In previous research, the dimensionality of the observing subscale has also shown an inconsequent item structure. While in the original KIMS one observing subscale was identified (Baer et al. 2004), a later analysis in a German sample revealed a two dimensional construct, i.e. observing of internal and external phenomena (Hoefling et al., 2011).

With regard to previous research, which treated mindfulness and decentering one-dimensionally and combined them into one single variable (Carmody et al. 2009), disagreeing results were obtained in the present study. These may be due to different methodological approaches in the investigation of the research task. Whereas in the study by Carmody and colleagues ( 2009) researchers applied the Baron and Kenny ( 1986) causal steps approach to mediation, the present study focused on testing mediation effects simultaneously in a structural equation framework, which is more susceptible to detect effects (Preacher and Hayes 2008). Furthermore, Carmody and colleagues ( 2009) aggregated the different aspects of mindfulness into one variable, whereas in the present analysis mindfulness was measured multidimensionally. Nevertheless, there is growing evidence in favor that the underlying factor structure is rather multidimensional (Baer et al. 2004; Baer et al. 2006; Hoefling et al. 2011). This multidimensionality has also been demonstrated in the present study by pointing out that the several components of mindfulness have a differential impact on decentering as well as on depression and, therefore, can be treated as distinct dimensions.

### Limitations and future research

The present results are based on cross-sectional data, however, the mediation model analyzed in the present study is based on a theoretical model derived from former empirical studies (e.g., Carmody et al. 2009; Teasdale et al. 2002). While this approach is common practice, future research, nevertheless, should assess the causal relations included in the present mediation model in a longitudinal research framework to foster their statistical and clinical significance. Regarding the clinical significance and generalization of the present results, another limitation refers to the present study sample. While the relationship between mindfulness and decentering was examined for its effects on a clinical variable, i.e., depressive symptoms, the study was conducted within a non-clinical sample. However, in this first step of this mediation analysis we regard a non-clinical sample as a suitable starting point, but we suggest investigating the mediation model in a clinical sample in future studies regarding the importance of mindfulness and decentering for good mental health. Therefore, it should be clarified whether the relationship between mindfulness and decentering is different within a clinical, e.g., depressive, sample compared to a healthy one. A second limitation related to the generalization of the present findings to other populations refers to the composition of the present study sample of higher educated and mainly Caucasian younger adults. Accordingly, the present study should be replicated within a different, e.g., older sample with mixed socio-economic status and composed of participants with varying ethnical backgrounds. Furthermore, prospective longitudinal studies might include mindfulness meditation practices into their settings in order to investigate the association between practicing mindfulness and the development of different mindfulness skills in relation to the development of better decentering abilities.

In the present mediation analysis, we have explored only one of the proposed working mechanisms of mindfulness, namely decentering. Thus, future investigations should clarify the associations between depression, mindfulness, decentering, and the additional mechanisms of change, e.g., values clarification, as proposed by Shapiro and colleagues ( 2006). Another conceptual consideration relates to the concept of detached mindfulness (Wells 2005). It would be interesting to investigate how this specific form of mindfulness fits into the multidimensional description of mindfulness. More precisely, which particular dimension of mindfulness can be elucidated by the concept of detached mindfulness and which role does decentering play within this context.

## Conclusion

In conclusion, the present data cannot support research converting decentering and mindfulness into one single factor, but rather suggests to treat them as two separable but related concepts. Specifically, decentering seems to act as a mediator delivering the salutary effects of mindfulness meditation practices on the severity of depressive symptoms. It was shown that Accepting without Judgment and Describing of observed phenomena both influence the depression severity directly, but that these two mindfulness skills also exert their influence on depression via the pathway of decentering. Furthermore, no direct relationships of Acting with Awareness and the Observation of internal phenomena on depressive symptoms could be demonstrated, but their effect has been shown to be delivered indirectly by the mediator decentering.
